# Third Eye? The Assistance of Artificial Intelligence (AI) in the Endoscopy of Gastrointestinal Neoplasms

**DOI:** 10.3390/jcm12216721

**Published:** 2023-10-24

**Authors:** Magdalena Leśniewska, Rafał Patryn, Agnieszka Kopystecka, Ilona Kozioł, Julia Budzyńska

**Affiliations:** 1 Students’ Scientific Circle on Medical Law at the Department of Humanities and Social Medicine, Medical University of Lublin, 20-093 Lublin, Poland; lesniewska.w.magdalena@gmail.com (M.L.); aga.kop@interia.eu (A.K.); ilona.koziol9@gmail.com (I.K.); julciab42@gmail.com (J.B.); 2 Department of Humanities and Social Medicine, Medical University of Lublin, 20-093 Lublin, Poland

**Keywords:** artificial intelligence, endoscopy, gastrointestinal tract, gastric cancer, colorectal cancer, esophageal cancer

## Abstract

Gastrointestinal cancers are characterized by high incidence and mortality. However, there are well-established methods of screening. The endoscopy exam provides the macroscopical image and enables harvesting the tissue samples for further histopathological diagnosis. The efficiency of endoscopies relies not only on proper patient preparation, but also on the skills of the personnel conducting the exam. In recent years, a number of reports concerning the application of artificial intelligence (AI) in medicine have arisen. Numerous studies aimed to assess the utility of deep learning/ neural network systems supporting endoscopies. In this review, we summarized the most recent reports and randomized clinical trials regarding the application of AI in screening and surveillance of gastrointestinal cancers among patients suffering from esophageal, gastric, and colorectal cancer, along with the advantages, limitations, and controversies of those novel solutions.

## 1. Introduction

Cancers of the gastrointestinal tract, especially of the stomach and large intestine, are a frequent cause of death due to malignant neoplasms. For this reason, there is a need to develop new technologies supporting diagnostics, mainly endoscopic methods in detecting gastrointestinal cancers [1]. The gold standard examination for reducing morbidity and mortality in colorectal cancer (CRC) is a colonoscopy with the detection and removal of adenomatous polyps [2,3,4]. However, in order to reduce morbidity and mortality in cancers of the upper gastrointestinal tract, endoscopic methods are used, such as: confocal laser endomicroscopy and narrow-band imaging [5,6,7,8]. Nevertheless, the method largely depends on the experience of the doctor performing the examination.

**Figure 1:**
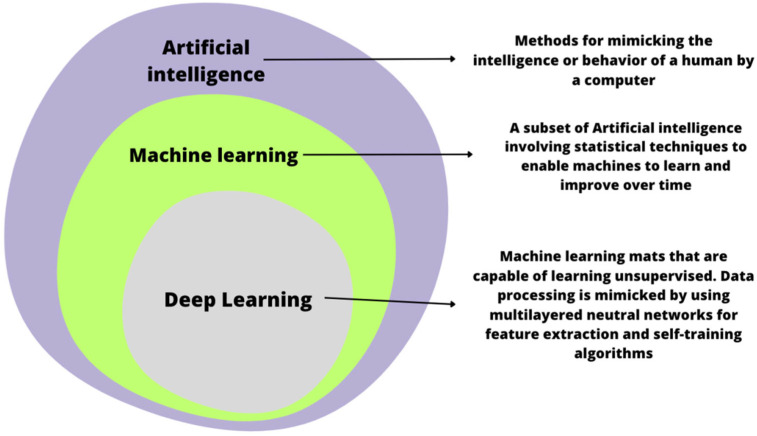
A comparison of artificial intelligence, machine learning, and deep learning.

In recent years, the application of artificial intelligence (AI) in the medical field has expanded [9]. The use of artificial intelligence can support classical methods by reducing the percentage of overlooked changes [1,10]. AI-assisted endoscopy, which uses facial recognition technologies based on AI, can detect abnormal conditions quickly, based on the analysis of colorectal images, thereby reducing the need for nontumor polypectomy [11]. Furthermore, computer-aided endoscopy has a high degree of accuracy and sensitivity, indicating potential applications in early CRC diagnosis. AI technology has rapidly developed over the past few years, allowing us to develop a computer-based screening system [12]. Artificial intelligence (AI)-powered endoscopic systems are supporting the clinician in various ways. They can detect and classify lesions with image and video analysis (computer vision algorithms). Machine learning algorithms may be helpful in the interpretation of the data collected during the exam. However, real-time assistance is also possible, such as guiding and providing information about screened colon tissues [13]. The use of artificial intelligence in endoscopic examinations of the gastrointestinal tract may help in distinguishing benign, non-cancerous lesions from neoplastic lesions. The use of artificial intelligence in endoscopic examinations of the gastrointestinal tract may help in distinguishing benign, non-cancerous lesions from neoplastic lesions [1] (Figure 1).

The aim of this narrative review was to summarize the most recent findings of clinical trials and to propose further directions in the research regarding the utility of modern AI technologies in the diagnostic and therapeutic processes of gastrointestinal neoplasms.

## 2. Esophageal and Gastric Cancer

Esophageal cancer and gastric cancer are some of the most common malignant tumors in the world. Characterized by high mortality rate, they are a major financial burden on health care systems. Detection of early gastric cancer and precancerous lesions is based on an endoscopic examination. One of the most common methods of gastroscopy is called white light gastroscopy. Early detection of suspicious lesions is important in extending survival [14,15,16,17,18]. Due to latent and non-specific symptoms, upper gastrointestinal cancer is usually detected at advanced stages, leading to a poor prognosis. But, if detected early, five-year survival can be up to 90% [19,20,21].

### 2.1. Esophagogastroduodenoscopy

Esophagogastroduodenoscopy (EGD) is a basic examination that visualizes the inside of the stomach. However, the subjective assessment and experience of the endoscopist influence the error rate, estimated at 20–40% in the case of early gastric cancer (EGC). Therefore, detailed guidelines for upper gastrointestinal endoscopy would standardize the results of these studies [14,22,23]. In the case of early gastric cancer, which is characterized by subtle changes that are difficult to see by an inexperienced endoscopist, it is essential to image the entire stomach to exclude EGC and avoid imaging blind spots [24,25].

High definition endoscopy is recommended as a gold standard in screening and also as a monitoring tool for Barrett’s esophagus, which is the most common precancerous condition of the esophagus [26,27,28]. Narrow-band imaging (NBI) is also a frequently used method in the diagnosis of the esophagus; additionally, targeted biopsies taken with this method resulted in the detection of a greater number of dysplastic areas, which in turn could reduce the number of samples taken [29].

### 2.2. Comparison of AI with EGD

Lianlian Wu et al., developed an AI-based system to analyze EGD images using a special grid model for the stomach to indicate the existence of blind spots on raw EGD films. Based on 200 gastroscopic images with or without lesions, DCNN correctly diagnosed the tumor with 92.5% accuracy, a 94% sensitivity, and a 91% specificity. Endoscopists of varying experience achieved rates ranging from 81.2% to 89.7%. Thus, the accuracy with DCNN was significantly higher than that of endoscopy specialists [25]. In a study by Niikura R et al., comparing endoscopic methods with those using artificial intelligence, it was shown that gastric cancer was diagnosed in 49 out of 49 patients (100%) in the group using AI and in 48 out of 51 (94.12%) in the group diagnosed using standard endoscopy. The situation was similar in the case of early gastric cancer, where AI diagnosed it in 100% of patients and endoscopy in 88.46% of patients. A significant increase in diagnosis of gastric cancer per image, defined as the number of images analyzed to determine a diagnosis, was observed in the AI group (747 out of 748 images, 99.87%) compared to the expert endoscopist group (693 out of 786 images, 88.17%). However, the intersection-over-union (IOU) gastric cancer diagnosis, explained as the amount of overlap between predicted areas and gold-standard borderlines, was lower (0.842) in the AI group than in the specialist endoscopist group (0.972) [10]. Similarly, a study by Gong EJ et al. all aimed to establish and validate a DCNN-based automatic detection and classification of gastric tumors in real-time endoscopy. DCNN-assisted endoscopy showed a higher lesion detection rate in DCNN-assisted endoscopy than in classical endoscopy. However, these results were not statistically significant (2.0% vs. 1.3%; *p* = 0.21) [30]. A study using an artificial intelligence system called ENDOANGEL-LD with the ability to detect gastric cancer in China found that the rate of unrecognized cases of gastric cancer was lower in the group of patients tested with AI (6.1%) compared to routinely performed endoscopy (27.3%) [14] (Table 1). There is evidence of the effectiveness of the DCNN-based method in the diagnosis of gastric cancer. This method may have an advantage in detecting early gastric cancer, but there is still a need for more knowledge and more clinical trials on a large group of patients.

### 2.3. Advantages of AI in Gastric and Esophageal Cancer Diagnosis

A randomized, prospective study enrolled 133 patients with Barrett’s esophagus (BE). There were two groups of participants: one received IRIS-enhanced virtual chromoendoscopy first (IRIS-VLE), followed by unenhanced VLE, while the other received the reverse sequence (VLE-IRIS). The unenhanced VLE assessment after the first IRIS review was found to be less time-consuming. Furthermore, 100% of the dysplastic areas of interest (ROIs) were identified with this test setup in comparison to 76.9% found in unenhanced VLE. A significant difference was not found between VLEs that had been enhanced with IRIS and those that were not [17]. In a pilot study of 65 patients with early esophageal neoplasia in BE as well as high-grade dysplasia or T1 cancer, the DCNN algorithm was used to detect dysplasia changes and draw boxes around these areas. In an analysis of 458 test images (225 with dysplasia and 233 without dysplasia), the system correctly detected early cancer with 96.4% sensitivity, 94.2% specificity, and 95.4% accuracy [32].

The advantage of using AI in the diagnosis of gastric polyps using the SSD-GPNet system is an increase in the mean average precision (mAP) of detecting gastric polyps by 2%, while the examination time is slightly longer. This method may be useful for real-time detection of gastric polyposis with less risk of false negative test results, which may be due to human factors [31]. In a study of 1099 patients with gastritis with or without *H. pylori* eradication, a machine learning (ML) model was used to predict gastric cancer risk in a personalized way. It allows for the creation of an individual follow-up strategy after the initial EGD in accordance with the risk assessment of gastric cancer [33]. One of the first computer-aided diagnostics (CADx) systems to identify irregular microvessels in EGC images used the gray-level co-occurrence matrix (GLCM). A magnifying narrow-band imaging (M-NBI) system was able to recognize EGC with an accuracy of 96.3%. Results were based on 147 patients, including 127 who had EGC [24].

One of the largest studies using the GRADIDS AI system for the diagnosis of cancer of the upper gastrointestinal tract, conducted in six hospitals with various degrees of reference, confirmed that the method can improve the skills of all endoscopists to the level of experts in this field. In addition, the increased sensitivity of the GRAIDS system in detecting neoplastic lesions may help in the earlier detection of gastric cancer and, consequently, reduce the high cost of treatment of cancers of the upper gastrointestinal tract [1]. In a study by Lianlian Wu et al., it was shown that the time to diagnose gastric cancer with DCNN was shorter than with standard diagnosis by an endoscopist. Shorter screening time, and lack of fatigue and the influence of subjective judgment, depending on the experience of endoscopists, may facilitate EGC diagnostics, enabling it to be carried out online. This system was also enriched with CAM or weighted linear sum of the presence of visual patterns with different characteristics and a grid model for the stomach to exclude the presence of dead spots in the stomach [25]. Another study tested deep learning networks for use in differentiating gastric ulcer and cancer through lesion classifications. A total of 200 healthy cases, 367 cancer cases and 220 ulcer cases were included in the study, and inception models, ResNet and VGGNet pre-trained in ImageNet were used. Cases involving a healthy stomach image, that is, normal vs. ulceration and normal vs. cancer, were compared, with an accuracy greater than 90%. While the differentiation between ulcer and cancer was recognized with an accuracy of 77.1%, this may be related to the smaller difference in the appearance of the stomach [34].

### 2.4. Disadvantages of AI in Gastric and Esophageal Cancer Diagnosis

The positive predictive value (PPV) of the GRAIDS system, which uses AI to diagnose the upper gastrointestinal tract, was lower compared to the opinion of real-time endoscopists. The GRAIDS system has the ability to recognize normal structures, for example, the pylorus, angle of the stomach and mucus, as well as the raised stomach wall during peristalsis, which may result in an increase in false-positive results. These normal structures would be easily recognized by the endoscopic physician and probably, in clinical practice with real-time GRAIDS, the false positive rate would be lower [1].

## 3. Colorectal Cancer

Colonoscopy is the most frequently employed screening method for CRC in the United States [35,36]. This exam is characterized by a high level of operator variability [37]. According to a meta-analysis, 26% of adenomas were missed [38,39]. Furthermore, the adenoma detection rate (ADR) is regarded as one of the most crucial quality metrics for assessing the effectiveness of colonoscopy. A colonoscopy requires a biopsy of every polyp; however, it is difficult in clinical practice. An alternative, simpler quality indicator called polyp detection rate (PDR) can be used as a good proxy for ADR [40,41], increasing the detection rate of adenoma by 1% [42]. Clinical approaches founded on the optical diagnosis of diminutive (5 mm) colorectal polyps have the potential to yield significant economic and financial benefits [43,44,45]. A total of 84% of European endoscopists report that they do not use the “leave-in-situ” and “resect-and-discard” strategies for fear of errors in optical diagnostics [43,46,47]. There are two most important factors affecting the percentage of incorrect diagnoses: blind spots and human error. It is feasible to mitigate blind spots, for instance, through the utilization of a wide-angle range or wide-angle remote attachment. However, addressing human fallibility proves to be a considerably more challenging task [48,49,50].

ADR varies among physicians, and tandem colonoscopy studies have shown that adenoma miss rates (AMR) can range from 6% to 41% [42,49,51,52]. As a result of the new CADe (computer-aided detection) system (GI-Genius; Medtronic, Minneapolis, Minnesota), an additional display is no longer necessary to display the AI detection field on endoscopic images, making it fully integrated into the endoscopy process, allowing real-time video processing at the same frame rate as the standard procedure, without any artificial modifications required to the colonoscopy procedure as usual [53]. It has been proven that for every 1.0% increase in the adenoma detection rate (ADR), there is a 3.0% reduction in the risk of intraoperative CRC [42,53,54]. However, polyps can be overlooked, with a miss rate of up to 27% due to the characteristics of both the polyps and the operator [49,50,53]. Several studies have shown that the assistance of a second observer increases the PDR; however, this strategic option is still controversial in terms of increasing the ADR [53,55,56,57,58] (Table 2).

### 3.1. Lynch Syndrome (LS) and Endoscopy

The Lynch syndrome (LS) is the most prevalent hereditary CRC syndrome resulting from pathogenic variants in DNA mismatch repair genes (MMRs) [69]. LS patients should be screened every 1–2 years with high-definition white-light endoscopes (HD-WLE) to detect small lesions, as recommended by the National Comprehensive Cancer Network [70]. It has been reported that from 12% to 62% of adenomas in LS are missed during colonoscopy, and specifically, small lesions and flat adenomas, which are characteristic of LS, are frequently missed [38,71]. In a randomized trial, 96 patients with LS were tested for adenoma detection rate (ADR) in HD-WLE with real-time AI (CADEYE) (50 patients) and without (46 patients). In the HD-WLE arm, adenomas were detected in 12/46 patients with ADR (26.1%) versus 18/50 patients in the AI arm with ADR (36.0%). In AI-assisted colonoscopies, 0.60 adenomas were detected per procedure, compared to 0.43 in conventional colonoscopies. Within the AI group, a larger percentage of the identified adenomas exhibited a completely flat morphology (56.6% vs. 20% (*p* = 0.018)). The utilization of AI-assisted colonoscopy in real-time holds promise for enhancing endoscopic surveillance in Lynch syndrome patients, especially in terms of improving the detection of flat lesions [72].

### 3.2. Adenoma Detection Rate (ADR)

The aim of Wang P. et al.’s work was to assess the effectiveness of a real-time automatic polyp detection system in enhancing the detection rates of polyps and adenomas within real clinical contexts. Their study demonstrated that the CADe system, founded on deep learning, produced a notable boost in the detection rates of colorectal polyps and adenomas in a region known by a low ADR prevalence. Due to its high precision and reliability, the current CADe system exhibits promising potential for integration into contemporary clinical practice to enhance the detection of colonic polyps [53]. A randomized trial was conducted with colonoscopists in their qualifying period (AID-2). Within the age range of 40–80, a total of 660 patients underwent high-resolution colonoscopies, administered by 10 non-expert endoscopists, each with less than 2000 colonoscopies to their credit. These procedures were conducted with or without real-time deep learning computer-assisted detection (CADe), using the GI Genius system from Medtronic. The results showed that in this cohort with evenly distributed study parameters, the collective ADR was notably higher in the CADe group compared to the control group (53.3% vs. 44.5%). Similar increases were observed in the number of adenomas and in small and distal lesions. It has been demonstrated that AI can enhance the ADR, serving as the primary surrogate endpoint for assessing colonoscopy quality. [63]. Other analyses presented by Yao L. et al. compared the results of four groups: the control group; the standard colonoscopy video, CADe group; the detected polyp location signalized with a hollow blue box, CAQ; the real-time withdrawal speed with a dashboard, COMBO; and a combination of CADe and CAQ. The ADR exhibited a significant increase in both the CADe and CAQ groups. However, it was notably higher in the COMBO group compared to the CADe group. No significant differences were observed between the CAQ and COMBO groups [60]. This suggests the importance of proper withdrawal time as a factor deciding of the final ADR. However, the authors confronted those results with previous literature reports. Some of them indicate that there is a correlation between the lesion detection and withdrawal time, while some of them contradict it [73,74,75]. A study conducted in Italy examined data from 685 patients who underwent screening colonoscopies for colorectal cancer (CRC), follow-up examinations after polyp removal, or investigations prompted by positive stool immunochemistry or signs/symptoms of CRC. These tests were conducted using the CADe system. The adenoma detection rate (ADR) was notably higher in the CADe group (54.8%) than in the control group (40.4%). Additionally, the number of adenomas detected during colonoscopy was significantly greater in the CADe group compared to the control group. Adenomas 5 mm or smaller were detected in a significantly higher percentage of people in the CADe group (33.7%) than in the control group (26.5%), as were adenomas 6 to 9 mm (detected in 10.6% of people in the CADe group vs. 5.8% in the control group), irrespective of the morphological characteristics and the location. No significant difference in withdrawal time was observed between the groups. The inclusion of CADe in real-time colonoscopy was found to significantly increase the ADR and detection of adenomas during colonoscopy without increasing the withdrawal time [65].

### 3.3. Polyp Detection Rate (PDR)

The prospective, single-center randomized trial had the objective of comparing PDR and ADR in colonoscopies. The exams of the 400 patients were included. The exclusion criteria comprised the following: prior experience of a colonoscopy, a medical diagnosis of inflammatory bowel disorders, a hereditary polyposis syndrome or colorectal cancer diagnosis, or a history of prior colorectal surgical procedures. Other circumstances such as existing contradictions for polypectomy or bad bowel preparation were also an exclusion criterion. Performed colonoscopies, which were incomplete, were not taken into account in the primary analysis. Study participants were divided into two groups. Exams were performed by two endoscopists—one with more than 15 years’ experience and one with 8 years’ experience. The analysis of exams performed in the morning and afternoon aimed to check the effect of the examiners’ fatigue on the PDR and ADR. No statistically significant disparities between the control and study groups were identified. Nevertheless, both in the morning and during the afternoon hours, the PDR and ADR demonstrated higher values when AI systems were employed [13]. A marginally statistically noteworthy augmentation in PDR with the use of computer-aided detection (CADe) (85.7%) in comparison to the control group (79.7%) was found in a study by Ahmed et al., and there was no statistically significant difference in ADR among those two groups [59]. One study used a new computer-aided diagnostic system (CAD-EYE; Fujifilm Co., Tokyo, Japan) to provide real-time polyp characterization using standard endoscopy. This study was designed to evaluate whether real-time artificial intelligence (AI) optical diagnosis by endoscopists with varying backgrounds is accurate enough to implement a leave-in-situ strategy for small (≤5 mm) rectosigmoid polyps (DRSPs). The accuracy of AI-assisted optical diagnostics was significantly lower for non-experts (82.3%) than for experts (91.9%). Non-experts have moved closer to the level of performance of more advanced people over time. Optical diagnostics supported by artificial intelligence meets the required PIVI (preservation and incorporation of valuable endoscopic innovations) thresholds, but the high level of experience and knowledge of endoscopists still plays an important role [43]. Almost 2500 patients were screened at six centers in the study and were referred for screening, follow-up, and diagnostic colonoscopy. Patients were randomized into a study group with AI-assisted colonoscopy (n = 1240) and a control group with standard colonoscopy (n = 1248). To compare the outcomes of the two groups, data were gathered and assessed in real-time. The final analysis was performed after exclusion of patients with suspected severe bowel disease and unqualified colonoscopy (research cohort 1177, comparison group 1175). Within the study cohort, there was a noteworthy increase in the detection of non-first polyps per colonoscopy (PPC-plus). However, there was no statistically significant rise in the PDR observed. Similar to findings in previously described research, the AI system exhibited a higher capacity for detecting flat polyps (5.9% vs. 3.3%). In the study cohort, in comparison to the control group, the detected polyps were more inclined to be diminutive polyps (76.0% vs. 68.8%) and less inclined to be small polyps (15.1% vs. 21.4%) [76].

In the Misawa, Masashi et al. study, the performance of the developed computer-aided detection (CADe) system was assessed. For this purpose, they retrospectively collected 73 colonoscopy films from 73 patients with a total of 155 colorectal polyps. The gold standard for the presence of polyps was the annotation of two expert endoscopists who viewed these recordings. CADe detected 94% of the polyps tested, while false positives were 60%. The AI accounted for 64.5% of flat polyps, which are generally considered difficult to pick out. In this study, the CADe sensitivity was 90%, the specificity was 63.3%, and the accuracy for the frame-based analysis was 76.5% [77]. In a different investigation, the AI system demonstrated a notable enhancement in PDR (*p* < 0.001), while AI-aided colonoscopy led to an elevated detection rate of polyps smaller than 6 mm (*p* < 0.001). However, no distinction was observed for larger lesions. It has been proven that, mainly in the case of smaller polyps, a real-time automatic polyp detection system can increase the PDR [48].

### 3.4. Adenoma Miss Rate (AMR)

The AMR was assessed in a study by Wang et al. The study group consisted of patients randomly assigned to one of the following paths: first, routine colonoscopy followed by a CADe examination, or second, primarily assessed with CADe and then evaluated with a routine colonoscopy. The authors report that the AMR was significantly lower with CADe colonoscopy (13.89%) than with routine colonoscopy (40.00%), especially in nonpedunculated types [16]. In one study, 280 patients were screened or followed up with CRC and randomized to undergo two one-day colonoscopies whether or not AI and vice versa. AMR (adenoma miss rate) was 15.5% and 32.4% in the AI and non-AI groups, respectively. In detail, the AMR was lower for AI first for lesions ≤ 5 mm and non-polypoid lesions and was lower in both the proximal and distal colons. In the group with the first colonoscopy, the mean number of adenomas at the second colonoscopy was lower compared to the first colonoscopy without AI. There were 6.8% false-negatives in the AI arm and 29.6% in the non-AI arm. Artificial intelligence led to an approximately 2-fold reduction in the error rate for colorectal cancer [64]. Glissen Brown JR et al. conducted a prospective, multicenter, tandem, randomized, single-blind study to evaluate the deep learning-based CADe system. A CADe or high-definition white light (HDWL) colonoscopy followed immediately by another tandem procedure by the same endoscopist was randomly assigned to colorectal cancer screening or follow-up patients. AMR was the primary endpoint, while SSL miss rate and APC were the secondary endpoints. AMR was lower in the CADe-first group compared to the HDWL-first group (20.12% vs. 31.25%). The SSL miss rate was lower in the first CADe group (7.14%) compared to the first HDWL group (42.11%). First-pass APC was higher in the CADe-first group. The ADR after the first pass was 50.44% in the CADe-first group and 43.64% in the HDWL-first group. A decrease in AMR and SSL miss rates and an increase in first-pass APC using the CADe system compared to HDWL colonoscopy alone have been demonstrated [51]. Another multicenter, randomized trial compared the AMS among two groups referred for screening and surveillance colonoscopy. A total of 179 patients were assigned either to receive a standard colonoscopy or a colonoscopy with computer-aided detection (CADe). The adenoma miss rate of the CADe was found to be significantly lower than that of the standard colonoscopy group (13.8% vs. 36.7%) [62].

A multicenter, randomized clinical trial included 894 screened patients and 465 CRC-supervised patients. The aim of the study was to determine whether CADe is an effective and safe method to support a standard colonoscopy. The patients were divided into a study group (438 screening tests, 244 observations) for examination performed with the CADe colonoscopy arm, and a control group—standard colonoscopy (456 screening tests, 221 observations). Comparing the study group with the control group, the number of APC examinations increased by 27%. No statistically significant decrease in true histology rate (THR) was obtained. Both screening and follow-up showed an increase in APC (28%) and 21%, respectively. The study group had an ADR increase of 3.9% that was not statistically significant [78]. In some cases, not all parts of the intestinal tract can be reached and examined, and such colonoscopy remains incomplete. The following procedure in those situations is a computer tomography colonography (CTC). It allows detecting structures occurring in the colon, but does not provide the possibility of harvesting tissue samples or seeing the morphology of the lesions. An alternative to computer tomography colonoscopy is a capsule endoscopy (CCE) [79,80,81]. Although harvesting tissue samples is also impossible, it allows a better analysis of the morphology of changes in the gastrointestinal tract. Capsule colonoscopy may be reinforced by the AI. This technique can be enhanced by incorporating artificial intelligence (AI) technology. AI can assist in determining the location of the camera during the examination, particularly in common scenarios where double recorded polyps occur due to the capsule’s backward movement [61].

### 3.5. Limitations and Controversies

One of the limitations of AI-aided endoscopy systems is false-positive detection, resulting in unnecessary polyp removals and longer examination times [13]. The others are the level of bowel preparation and the limitations of AI devices, the developed algorithms, sample size, and technical aspects (processors, etc.) [60,82].

Many reports show the value of AI in detecting suspicious lesions of the colon or reducing the rate of missed ones. Although this makes screening more efficient, it might impair the capability of young trainee endoscopists to rely on their skills. This issue was brought up by Sinagra et al. in a commentary [82]. According to the authors, future studies on AI in endoscopy should include endoscopists of various experiences, and AI-aided systems should be carefully implemented in training programs. There is a need for evidence-based guidelines for AI implementation, which would substantially increase physicians’ trust in these novel solutions.

## 4. Conclusions

AI’s role in the medical field grows, and new possibilities for the application of algorithms and real-time solutions arise. Most of them regarding the endoscopy exam are real-time support systems for marking suspicious lesions. For the correct use of the AI, it is required that the procedure meets quality standards with a correct exposure of all the mucosa. The majority of studies concerned patients randomized either to groups receiving CADe colonoscopy or standard colonoscopy as the first performed exam, which was followed by the other, or patients being examined by endoscopists with large or moderate experience. Future studies should check the PDR, AMR, and ADR among endoscopists with various experiences and exams with and without CADe support over a longer period of time.

A longer period of observation and multiple exams performed by the same personnel in comparison to exams performed with the support of AI would enable us to assess the average PDR, AMR, ADR of a human investigator and AI’s capabilities in detecting cancerous lesions. Such observations would finally give the answer, whether the AI is the “third eye” in cancer screening of the gastrointestinal tract.

The current challenge of AI is to integrate in the same team AI systems for upper endoscopy and low endoscopy with support systems in early lesion detection and endoscopic quality control at an affordable price for a greater number of endoscopic units.

## Figures and Tables

**Table 1:** Randomized controlled trials regarding the usage of AI in upper GI endoscopy.

Authors, Year	Study Groups	Endoscopy, AI Technology	Findings	Bibliography
Niikura R, et al., 2022	500 patients (249 were allocated to the AI diagnosis group and 251 to the expert endoscopist diagnosis group)	White light endoscopy, SKOUT; Iterative Scopes, Cambridge, MA	The per-image rate of gastric cancer diagnosis was higher in the AI group (99.87%, 747/748 images) than in the expert endoscopist group (88.17%, 693/786 images)	[10]
Lianlian, W et al., 2021	1812 patients (907 were allocated to the AI diagnosis group and 905 to the routine-first group, endoscopy group)	White light endoscopy, ENDOANGEL-LD system	The use of an AI system during upper gastrointestinal endoscopy significantly reduced the gastric neoplasm miss rate.	[14]
Zhang, X et al., 2019	215 patients	SSD for Gastric Polyps (SSD-GPNet)	SSD-GPNet improves polyp detection recalls over 10%	[31]
Kanesaka, T et al., 2018	147 patients (127 patients with EGC and 20 non-EGC participants)	Magnifying narrow-band imaging (M-NBI), computer-aided diagnostics (CADx)	96.3% EGC detection accuracy based on irregular microvessel imaging	[24]
Kahn, A et al., 2022	67 patients underwent virtual chromoendoscopy (VLE)-IRIS, 66 in the IRIS-VLE group	High-definition white light (HDWLE) and narrow-band imaging (NBI), intelligent real-time image segmentation (IRIS)	100% of dysplastic areas were identified when applying IRIS and 76.9% with VLE as the first interpretation modality (*p* = 0.06)	[17]

**Table 2:** Randomized controlled trials regarding the usage of AI in lower GI endoscopy.

Authors, Year	Study Groups	Endoscopy, AI Technology	Findings	Bibliography
Gimeno-García, AZ et al., 2023	400 (study group—2060, control group—194)	White light imaging, ENDO-AID CADe AI device, (Olympus Medical Systems, Tokyo, Japan)	higher PDR and ADR rate with the AI device (not statistically significant)	[13]
Ahmad, A et al., 2023	554 (study group—293, control group—286)	High—definition endoscopy, Endo- cuff Vision (Olympus, Tokyo, Japan) or a transparent plastic cap (Olympus) GI Genius system	Borderline statistically significant increase in PDR with the use of CADe in comparison to the control group	[59]
Wang, P et al., 2020	CADe + routine colonoscopy group (n = 184), routine colonoscopy + CADe group (n = 185)	White light endoscopy, EndoScreener, Shanghai Wision AI Co, Ltd., Shanghai, China	AMR significantly lower with CADe colonoscopy than with routine white-light colonoscopy (13.89% vs. 40.00%, *p* < 0.0001), PMR; lower with CADe colonoscopy than with routine white-light colonoscopy (12.98% vs. 45.90%, *p* < 0.0001), no statistical differences in the miss rate of advanced adenomas and SSAs/Ps.	[16]
Yao, L. et al., 2022	1120 participants divided into four groups: control group, CAD group, CAQ group, COMBO group	“EndoAngle”; real-time CADe model (Wuhan EndoAngel Medical Technology Company Co., Ltd., Wuhan, China), YOLO V3	ADR was higher in the COMBO group compared with the CADe group	[60]
Deding, U et al., 2020	97 patients, who underwent computed tomography colonoscopy (CTC) and colon capsule colonoscopy (CCE)	Olympus Evis Exera III 190^®^ (Olympus, Tokyo, Japan), PillCam2^®^, Medtronic, Minneapolis, MN, US	CCE has a higher sensitivity than CTC. The accordance of developed algorithm to the actual state was 77%	[61]
Kamba, S et al., 2021	179 patients in the standard colonoscopy group and 179 in the CADe as a first exam performed	White light imaging, YOLOv3, EVIS LUCERA ELITE (Olympus Medical Systems, Tokyo, Japan)	ADR of CADe first group was significantly higher than that of standard colonoscopy in the first pass of the SC-first group (*p* = 0.036)	[62]
Repici, A et al., 2022	660 patients divided into control group and CADe group	High-definition endoscopy, GI Genius, Medtronic, Minneapolis, MN, USA	Overall ADR was higher in the CADe than in the control group (53.3% vs. 44.5%)	[63]
Wallace, MB et al., 2022	230 patients randomized (1:1) to undergo 2 same-day, back-to-back colonoscopies with or without AI	White light colonoscopy, GI-Genius, Medtronic, Minneapolis, MN, USA	AMR was 15.5% in the arm with AI and 32.4% in non-AI colonoscopy first; AMR was lower for AI first for the ≤5 mm and nonpolypoid lesions and it was lower both in the proximal and distal colon	[64]
Luo, Y et al., 2021	150 patients divided into traditional colonoscopy group and AI-assisted colonoscopy group	White light endoscopy, real-time automatic polyp detection system Xiamen Innovision Co., Ltd., Jimei, Fujian, China, YOLO network architecture	AI system significantly increased the PDR (34.0% vs. 38.7%), AI-assisted colonoscopy increased the detection of polyps smaller than 6 mm	[48]
Repici, A et al., 2020	685 patients divided into groups who underwent high-definition colonoscopies with the CADe system or without (controls)	High-definition endoscopy, GI-Genius, Medtronic, Minneapolis, MN, USA	In the CADe group, the ADR was significantly higher than in the control group (40.4%), adenomas detected per colonoscopy were higher in the CADe group	[65]
Wang, P et al., 2019	1058 patients (536 standard colonoscopy, 522 colonoscopy with computer-aided diagnosis)	White light endoscopy, real-time automatic polyp detection system (Shanghai Wision AI Co, Ltd., Shanghai, China)	AI system significantly increased ADR (29.1% vs. 20.3%) and the mean number of adenomas per patient	[53]
Rondonotti, E et al., 2023	389 patients	Blue light imaging, real-time convolutional neural network-based AI system (CAD-EYE), Fujifilm Co., Tokyo, Japan)	AI-assisted high confidence optical diagnosis was made in 92.3%; The NPV (negative predictive value) of AI-assisted optical diagnosis for DRSPs (Preservation and Incorporation of Valuable endoscopic Innovations) was 91.0%	[43]
Glissen Brown, JR et al., 2022	232 patients (116 CADe colonoscopy first, 116 HDWL colonoscopy first)	High-definition white light (HDWL), Olympus CLV 190-series colonoscopes	AMR was lower in the CADe-first group compared with the HDWL-first group (20.12% vs. 31.25%); SSL miss rate was lower in the CADe-first group (7.14%) vs. the HDWL-first group (42.11%)	[51]
Yamada, A et al., 2020	15,933 (training images) 4784 (testing images)	Single Shot Multibox Detector for capsule endoscopic colon lesions detection	specificity 87.0% sensitivity 79.0%, accuracy 83.9%, AUC 0.902,	[66]
Gong, D et al., 2019	704 (study group—355, control group—349)	The ENDOANGEL system	ADR: ENDOANGEL group (58 (16%) of 355) vs. the control group (27 (8%) of 349), (odds ratio [OR] 2.30, 95% CI 1.40–3.77; *p* = 0.0010)	[67]
Wang, P et al., 2018–2019	962 (study group—484, control group—478)	computer-aided detection (CADe)	Adenomas detected: the CADe group (165 (34%) of 484) vs. control group (132 (28%) of 478)	[68]

## Data Availability

Not applicable.
